# A CRISPR-Cas9 based shuffle system for endogenous histone H3 and H4 combinatorial mutagenesis

**DOI:** 10.1038/s41598-021-82774-4

**Published:** 2021-02-08

**Authors:** Yu Fu, Zhenglin Zhu, Geng Meng, Rijun Zhang, Yueping Zhang

**Affiliations:** 1 Laboratory of Feed Biotechnology, State Key Laboratory of Animal Nutrition, College of Animal Science and Technology, College of Veterinary Medicine, China Agricultural University, Haidian District, Beijing, 100193 China; 2 School of Life Sciences, Chongqing University, No. 55 Daxuecheng South Rd., Shapingba, Chongqing, 401331 China

**Keywords:** Epigenetics, Genomic engineering, Histone analysis

## Abstract

Post-translational modifications of histone proteins greatly impact gene expression and cell fate decisions in eukaryotes. To study these, it is important to develop a convenient, multiplex, and efficient method to precisely introduce mutations to histones. Because eukaryotic cells usually contain multiple copies of histone genes, it is a challenge to mutate all histones at the same time by the traditional homologous recombination method. Here, we developed a CRISPR-Cas9 based shuffle system in *Saccharomyces cerevisiae*, to generate point mutations on both endogenous histone H3 and H4 genes in a rapid, seamless and multiplex fashion. Using this method, we generated yeast strains containing histone triple H3–K4R–K36R–K79R mutants and histone combinatorial H3–K56Q–H4–K59A double mutants with high efficiencies (70–80%). This CRISPR-Cas9 based mutagenesis system could be an invaluable tool to the epigenetics field.

## Introduction

In the nucleus of eukaryotic cells, nucleosomes are basic units of chromatin and play essential roles in DNA-templated processes, including packing, transcription, replication, recombination, and repair, together with organization and maintenance of chromatin structure. A nucleosome is composed of about 147 base pairs of DNA wrapped around a histone octamer, which is consist of two copies of core histones H2A, H2B, H3, and H4^1–3^. Core histones can be covalently post-translational modified by methylation, phosphorylation, acetylation, ubiquitylation, and sumoylation. These modifications impact gene expression by either directly altering nucleosome structure or recruiting histone modifiers^1,4–6^. Because there are multiple copies of histone genes in higher eukaryotic organisms, i.e. 100 copies of histone genes in *Drosophila melanogaster* and 64 copies of histone genes in human genome^7,8^, it is a challenge to generate histone site-directed mutagenesis to study histone modifications and related enzymes.

The budding yeast *Saccharomyces cerevisiae* has been served as a model organism to explore histone functions, because of several advantages (i) its highly-conserved histone sequences and modification functions with higher eukaryotes, (ii) its only two copies of histone genes in haploid cell stage, (iii) its ability to survive and grow with only one copy of histone genes, and (iv) its availability of powerful genetic manipulation tools. Several strategies have been carried out for systematic histone mutation libraries in *S. cerevisiae*: (1) transforming an episomal plasmid containing histone mutants with both endogenous copies deleted, (2) mutating one copy of endogenous histones by homologous recombination (HR) with the other copy deleted, (3) mutating both endogenous copies of histones by HR using two different selection markers^9–11^. These strategies have been very helpful to study histone functions and modifications, however additional requirements have emerged for new tools. Firstly, histone dosage effects have been discovered for some point mutations, thus previous identified crucial histone mutants by deleting one or two copies of endogenous histone may have different phenotypes with both endogenous copies mutated^11,12^. Secondly, multiple histone modifications have the same effect on gene expression or chromatin structure, and methods are needed for generating combinational mutagenesis on histones simultaneously. Last but not least, seamless mutagenesis is preferred for histone mutagenesis to reveal endogenous functions.

The recently developed CRISPR technology has greatly accelerated the speed of genome engineering^13–16^. CRISPR-Cas9 has also been implemented in histone mutagenesis in in a wide range of organisms, including human cell lines, a protozoan parasite *Trypanosoma brucei*, and *Drosophila melanogaster*^7,17–20^. However, there are several limitations for the current CRIPSR-Cas9 system: (i) CRISPR-Cas9 mutagenesis requires an efficient guide RNA (gRNA) targeting sequence and a following “NGG” as Protospacer Adjacent Motif (PAM) which are closed to the mutation sites; however, some targeted mutation sites do not meet such conditions. (ii) The current CRISPR-Cas9 systems performed single histone site mutation and are not capable to generate combinatorial histone mutations. In this paper, we developed a CRISPR-Cas9 based shuffle system for endogenous histone H3 and H4 combinatorial mutagenesis in *Saccharomyces cerevisiae*. Using this system, seamless point mutations on histone H3 and H4 in different combinations can be generated simultaneously with high efficiencies.

## Results

### Generating CRISPR-Cas9 based shuffle strains in *Saccharomyces cerevisiae*

**Figure 1:**
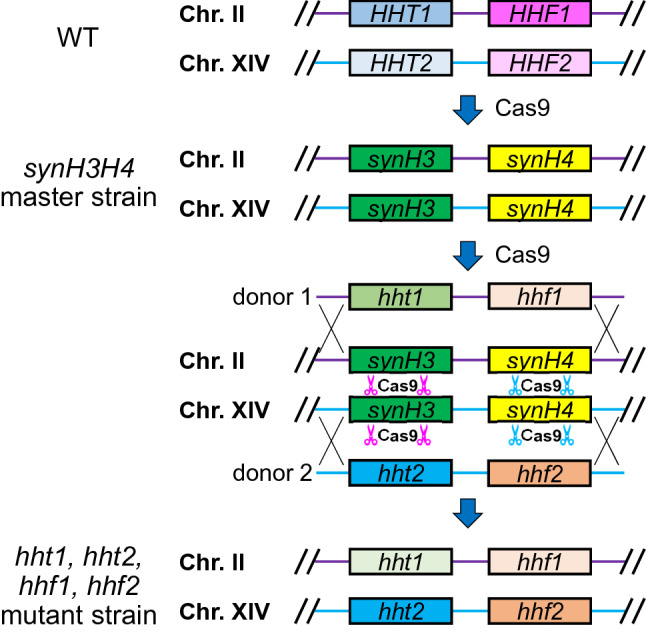
Graphic representation of CRISPR-Cas9 based shuffle system for endogenous histone H3 and H4 mutagenesis. As first step, synthetic H3 and H4 genes (*synH3* and *synH4*) are shuffled into genome by CRISPR-Cas9 to replace endogenous H3 and H4. Then the synthetic H3 and H4 genes are shuffled-out by CRISPR-Cas9 and repaired by donors with endogenous H3 and H4 genes with desired site-directed mutations.

**Figure 2:**
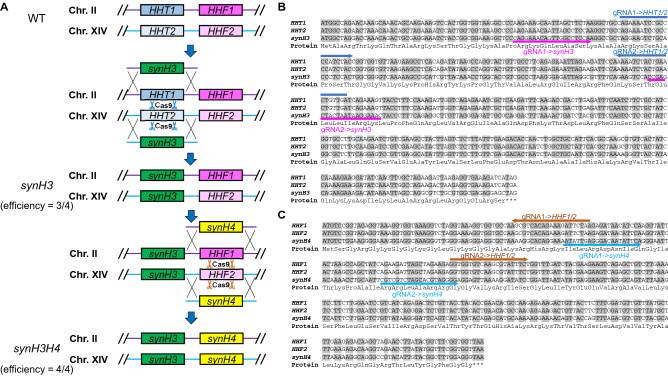
Generation of master shuffle strains with synthetic H3 and H4 by CRISPR-Cas9. (**A**) Generation of a master shuffle strain with synthetic H3 and H4 genes (*synH3H4*) through 2 rounds of CRISPR-Cas9 mediated gene replacements. The efficiencies of 2 rounds are 3/4 (75%) and 4/4 (100%) respectively, which are tested by four randomly picked colonies respectively. (**B**,**C**) The nucleotide sequence alignment of endogenous and synthetic H3 and H4. The synthetic H3 and H4 (synH3 and synH4) share same protein sequences but different DNA codons with native H3 and H4 genes. The nucleotides with grey background indicate the identical nucleotide sequences. Dark blue arrows represent gRNA sequences targeting endogenous H3 (*HHT1/2*); brown arrows represent gRNA sequences targeting endogenous H4 (*HHF1/2*); magenta arrows represent gRNA sequences targeting *synH3* genes, and light blue arrows represent gRNA sequences targeting *synH4* genes*.* The gRNAs targeting synthetic histones are used for generating Histone H3 and H4 mutants in Figs. 3 and 5.

In *S. cerevisiae*, the core histone genes are organized in H2A–H2B and H3–H4 gene pairs and driven by bidirectional promoters. *HHT1–HHF1* and *HHT2–HHF2* encode histone H3–H4 pairs on chromosome II and XIV respectively. Although histone H3 and H4 genes have corresponding identical amino acid sequences, they have 3% (8/309) and 5% (22/409) respectively differences in DNA coding and dramatically different upstream and downstream expression regulating elements. We designed a histone shuffle system for endogenous histone H3 and H4 combinatorial mutagenesis (Fig. 1). Firstly, a shuffle master strain was constructed, and endogenous H3 and H4 coding regions were replaced by synthetic H3 and H4 genes (*synH3* and *synH4*) by the CRISPR-Cas9 system. Then, *synH3* and *synH4* genes were targeted by CRISPR-Cas9 and repaired by PCR fragments amplified from endogenous H3 and H4 sequences with desired mutations. To minimize the chance of homology-directed repair at undesired sites, the *synH3* and *synH4* share same protein sequences but different codons with endogenous H3 and H4 (see sequence alignments in Fig. 2B,C).

The histone shuffle master strains with synthetic H3 and H4 (*synH3H4*) were generated by two steps of CRISPR-Cas9 mediated gene replacement (Fig. 2A). For the first step, endogenous H3 (*HHT1/2*) genes are replaced by the *synH3* gene. Cas9 plasmids containing 2 gRNAs targeting two loci of H3 genes and synthetic H3 PCR donors with 40–50 bp homologous recombination (HR) arms to the upstream and downstream of both *HHT1* and *HHT2* genes were electroporated to yeast CEN.PK 113-5D strain. 4 colonies have been randomly selected for verification, and 75% of colonies show correct gene replacement at both endogenous loci (Fig. 2A). For the second step, endogenous H4 (*HHF1/2*) genes are similarly replaced by the *synH4* gene with 100% efficiency (4/4 colonies) (Fig. 2A). In order to eliminate the possibility that the donor DNA fragments would be integrated into other chromosomal positions with random insertion, we performed whole genome sequencing of *synH3H4* strain. And the results showed *synH3* and *synH4* were integrated at desired loci without any detection of other integration loci (Supplementary data).

### Generation of histone H3 single and triple mutant through single transformation

**Figure 3:**
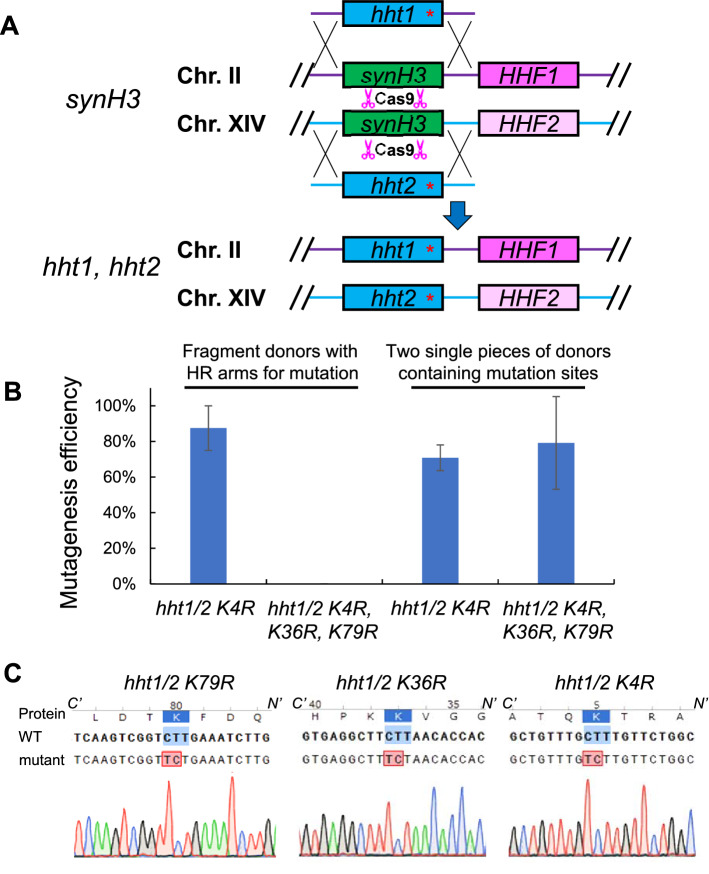
Generation of histone H3 single and triple mutants through a single transformation. (**A**) Graphic representation of CRISPR-Cas9 based mutagenesis on histone H3. The transformation was performed on *synH3* strain with 2 targeting gRNAs and donors harboring mutation sites and HR regions. The single piece donors were generated through fusion PCR. (**B**) The mutagenesis efficiencies for generating histone H3 single and triple mutant through transformation with different formats of donors. The repairing donors can be prepared by either multiple donor fragments with flanking HR arms for mutation or two single pieces of donors containing mutation sites. The data of bar charts represent mean averages of over-all mutagenesis efficiencies of 3 biological replicates with each randomly picked 8 colonies. The error bars indicate the standard deviations of 3 biological replicates. (**C**) The representation of sequencing results of both Histone H3 K4R–K36R–K79R at endogenous loci. The sequence alignments were performed by SnapGene software.

Histone H3 K4, K36, and K79 are the major methylation sites highly associated active gene transcription, and these modifications are conserved from yeast to human. To determine whether our system can be applied for histone single mutation, we sought out to generate histone H3 K4R mutant first. The repairing donors were prepared in two methods: two DNA PCR fragments for repairing and replacing each *synH3* gene with K4R mutation sites on the flanking HR arms (total four pieces for *hht1 K4R* and *hht2 K4R*) (Fig. 3A), and two single-piece donors containing mutation sites generated by fusion PCR (Fig. 3B). The donors and plasmids containing Cas9 and 2 gRNAs targeting *synH3* were transformed into the *synH3* strain. The correct gene replacement efficiencies of two strategies are at 88% and 71% with no significant difference (student T-test, *p* = 0.1161) (Fig. 3C, left). The two strategies for donor preparation were also tested for generating histone H3 K4R–K36R–K79R triple mutant. The efficiency of 79% was achieved by transforming two single-piece donors containing triple mutants, while the replacement efficiency of 0% was achieved by using 8-fragment donors with 40–50 bp homologues repairing arms (Fig. 3C, right). The low efficiency of the “fragment donors” strategy may be due to the unsuccessful homology direct repair (HDR) of the multiple-fragment donors.

**Figure 4:**
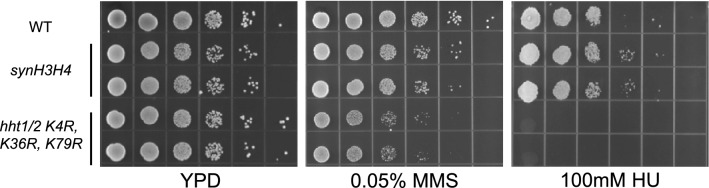
Confirmation of MMS and HU sensitivity of *synH3H4* strain and histone H3 K4R–K36R, K79R mutant. WT stain and two biological replicates of each mutant were grown in YPD overnight at 30 °C. Serial tenfold dilutions of OD_600_ = 1 yeast cultures were spotted onto YPD for 2 days, YPD with 0.05% MMS for 3 days, or YPD with 100 mM HU and incubated for 3 days at 30 °C.

To determine whether histone H3 triple mutant sensitive to methyl methanesulfonate (MMS) or hydroxyurea (HU) mediated DNA damage. Serial tenfold dilutions of OD_600_ = 1 were spotted onto YPD plate, YPD plate with 0.05% MMS, or YPD plate with 100 mM HU. And the results show *synH3H4* strains are not sensitive to 0.05% MMS or 100 mM HU, while *hht1/2 K4R–K36R–K79R* mutants are sensitive to 0.05% MMS and 100 mM HU (Fig. 4). These results demonstrated that the codon differences do not affect the shuffle master *synH3H4* strain sensitive to DNA damage, while the *hht1/2 K4R–K36R–K79R* mutants are sensitive as previously reported^21^.

### Mutagenesis on both histone H3 and H4 through single transformation

**Figure 5:**
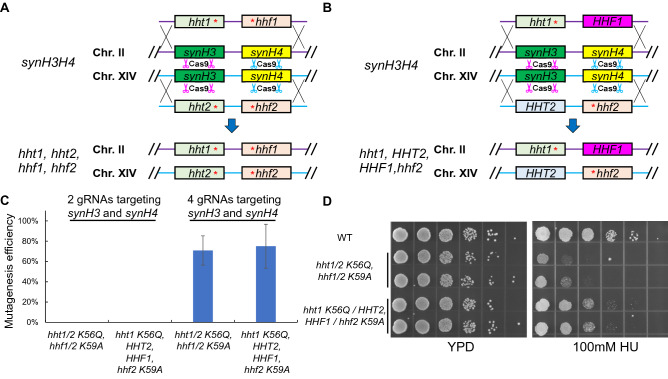
Mutagenesis on both histone H3 and H4 through single transformation. (**A**,**B**) Graphic representation of CRISPR-Cas9 based gene replacement for mutagenesis on both histone H3 and H4 with different combinations. By transforming different donors, the endogenous two copies of histone H3 and H4 can be repaired for identical mutants (**A**) or with different mutation sites (**B**). (**C**) The mutagenesis efficiencies for generating histone H3 and H4 mutants through single transformation with different gRNAs. 2 gRNAs: 1 gRNA targeting *synH3* and 1 gRNA targeting *synH4* and 4 gRNAs: 2 gRNAs for each synthetic histone. The data of bar charts represent mean averages of over-all mutagenesis efficiencies of 3 biological replicates with each randomly picked 8 colonies. The error bars indicate the standard deviations of 3 biological replicates. (**D**) Confirmation of HU sensitivity of histone H3 and H4 mutant. Two biological replicates were grown in YPD overnight at 30 °C. Serial tenfold dilutions were spotted onto YPD or YPD with 100 mM HU and incubated at 30 °C for 3 days.

We have demonstrated that the CRISPR-Cas9 based histone shuffle system is useful for histone H3 mutagenesis with high efficiencies. Next, we sought out to determine whether this system can be applied for mutagenesis on both histone H3 and H4 with different mutation combinations (Fig. 5A,B). Histone H3 K56Q mutant and histone H4 K59A mutant have been previously shown growth defects on medium containing 200 mM HU compared to wild-type strains^22^. We implemented our system for generating H3 K56Q and H4 K59A on both copies of histone genes or by combination of mutants and wild-type genes (Fig. 5A,B). We first transformed single-piece donors with Cas9 plasmid containing 2 gRNAs (One gRNA targeting *synH3* and the other targeting *synH4*). To our surprise, there was no successful gene replacement colony (Fig. 5C, left). This result suggested that either DNA cleavaged by Cas9 or HDR may not be efficient. To improve the chance of Cas9 cleavage, the Cas9 plasmid containing 4 gRNAs (with each histone synthetic gene targeted by 2 gRNAs, and gRNA sequences showed in Fig. 2B,C) was constructed and transformed with the same donors. With the help of additional gRNAs, the correct mutant efficiencies were improved to 71% for *hht1/2 K56Q, hhf1/2 K59A* mutant and 75% for *hht1 K56Q/HHT2, HHF1/hhf2 K59A* single-copy mutant*.* The HU sensitivity assay showed the mutations of *hht1/2 K56Q, hhf1/2 K59A* were sensitive to 100 mM HU, while the single-copy mutants *hht1 K56Q/ HHT2, HHF1/ hhf2 K59A* were less sensitive to 100 mM HU than double-copy mutants (Fig. 5D). These results demonstrated CRISPR-Cas9 based histone shuffle system is versatile for mutagenesis of histone H3 and H4 mutations with different combinations.

## Discussions

In this study, we developed a convenient, seamless, and efficient CRISPR-Cas9 based shuffle system to precisely introduce multiple mutations to histones H3 and H4 in *Saccharomyces cerevisiae*. We demonstrated this method by generating yeast histone triple H3–K4R–K36R–K79R mutants and histone H3–K56Q–H4–K59A double mutants with high efficiencies (70–80%). This system is also capable to mutate two copies of histones with any combinatorial mutations.

Although CRISPR-Cas9 has been applied to generate protein point mutations, it still needs to consider the limitation of gRNA closed to the desired mutation sites. Our method provides an alternative two-step method: firstly, replacing the original gene with a synthetic copy (codons changed) by CRISPR/Cas9, then targeting the synthetic copy using CRISPR/Cas9 and repair back to the endogenous gene with desired mutations.

We also found that increasing gRNA numbers to target a multiple-copy gene can improve gene replacement efficiencies. One possible explanation is that a single double strain break (DSB) site would be easily repaired by the other uncleavaged gene copies, while multiple DSB sites on single gene would trigger homology directed repair by donor DNA fragments when other gene copies also cleavaged. This finding can provide strategies for other multiple-copy gene editing.

For the future studies, it is useful to generate synthetic master strain with histone H2A, H2B, H3, and H4. Thus, any combinatorial mutations on histone genes can be generated through a single transformation.

## Material and methods

### Strains and broth media

*Escherichia coli* Top10 was used for vector cloning. The yeast strain used in this work was *Saccharomyces cerevisiae* CEN.PK 113-5D (*MAT a MAL2-8*^*c*^* SUC2 ura3-52*). *E. coli* top10 cells were grown on LB-Agar or LB broth with 50 mg/L Amp antibiotics for plasmid construction. Yeast strains were grown in YPD media with 2% glucose before the transformation. Transformants were plated on synthetic complete (SC) media minus uracil plates to select yeast cells. SC-5-FOA agar plates were employed to drop-out Cas9 plasmids.

### Plasmid construction

The *SynH3* and *synH4* coding sequences were designed according to the codon usage of S. cerevisiae, synthesized by Sangon Biotech (Shanghai) Co., Ltd., and cloned into pUC57 vector. The pCas vector with multiple gRNA was constructed as the previous report^23^.

### Donor DNA preparation

Donors used in this work were obtained by PCR reaction and purified by gel extraction. we cloned *HHT1–HHF1*, and *HHT2–HHF2* pair genes with their upstream and downstream sequences on pUC18 vectors. The repair donors were generated by PCR or fusion PCR with indicated primers amplification from the plasmid and purified by gel extraction.

### Yeast transformation and mutant identification

Yeast transformation was carried out using the electroporation as the previous report^23^. Genome DNA of strains survived on the auxotrophic plate was extracted for PCR reaction. The genotype of H3 and H4 of mutants were identified with PCR amplification and followed DNA sequencing. And genome DNA of *Saccharomyces cerevisiae* CEN.PK 113-5D was used as a control. H3 and H4 sequences of mutants were amplified by primers on ~ 100 bp upper and lower sites for sequencing analysis. The mutation rate was calculated as the ratio of mutants to total colonies tested with 3 biological replicates.

### DNA damage sensitivity assays

WT stain and two biological replicates of each mutant were grown in YPD overnight at 30 °C. Yeast culture with diluted at OD_600_ = 1 as the starting point. Serial tenfold dilutions of yeast culture were spotted onto YPD for 2 days, YPD with 0.05% MMS for 3 days, or YPD with 100 mM HU and incubated for 3 days at 30 °C.

## Supplementary Information

Supplementary Information
