# Supplementary material for: A CRISPR-Cas9 based shuffle system for endogenous histone H3 and H4 combinatorial mutagenesis
Source: Sci Rep. 2021 Feb 8;11:3298. doi: 10.1038/s41598-021-82774-4 (PMC7870972; doi:10.1038/s41598-021-82774-4)

Supplementary data for

**A CRISPR-Cas9 based shuffle system for endogenous histone H3 and H4 combinatorial mutagenesis**

Yu Fu^1^, Zhenglin Zhu^2^, Geng Meng^1^, Rijun Zhang^1*^, Yueping Zhang^1*^

^1^ Laboratory of Feed Biotechnology, State Key Laboratory of Animal Nutrition, College of Animal Science and Technology, College of Veterinary Medicine, China Agricultural University, Haidian District, Beijing 100193, China.

^2^ School of Life Sciences, Chongqing University, No. 55 Daxuecheng South Rd., Shapingba, Chongqing, 401331, China

Corresponding authors: R.Z., [zhangrj621@126.com](mailto:zhangrj621@126.com); Y.Z., [zhangyueping@cau.](mailto:zhangyueping@cau.c)edu.cn.

Sequences of *synH3* and *synH4*

***synH3*：**

5’-ATGGCTAGGACCAAACAGACTGCGAGGAAATCTACCGGCGGAAAGGCTCCCAGGAAACAGTTGGCCTCCAAGGCCGCTAGGAAGTCTGCACCCTCCACTGGCGGGGTCAAAAAGCCGCATCGTTACAAACCTGGCACCGTCGCCCTAAGGGAGATTAGGCGTTTTCAGAAGTCCACCGAGCTACTAATAAGGAAACTACCCTTTCAGAGGCTTGTTAGGGAGATTGCGCAGGACTTTAAAACTGATCTACGTTTCCAGTCCTCCGCTATTGGCGCTCTTCAGGAGTCCGTTGAGGCATATTTGGTTTCCTTGTTCGAGGACACTAATCTAGCTGCAATCCATGCTAAAAGGGTTACCATACAGAAGAAAGACATAAAATTAGCCAGGCGTCTGCGTGGCGAGAGGAGCTAG-3’

***synH4*：**

5’-ATGTCTGGAAGGGGAAAGGGAGGCAAAGGGTTGGGGAAGGGAGGCGCTAAAAGGCACAGGAAAATATTGAGGGACAATATTCAGGGAATTACAAAACCTGCAATTCGTCGTCTAGCACGTAGGGGAGGAGTTAAAAGGATATCCGGATTAATATATGAGGAAGTTAGGGCAGTATTAAAGTCATTTCTTGAGTCTGTTATAAGGGATTCAGTCACATATACAGAGCATGCAAAAAGGAAAACAGTCACCAGTTTAGACGTCGTCTACGCATTAAAAAGGCAGGGCCGTACATTGTACGGCTTTGGAGGGTAA-3’

**Lists of Primers**

**Supplementary table S1. Primers for Construction of Sub-cloning Vectors and Donor DNA**

| **Primer** | **SeQUENCE** | **Function** |
| --- | --- | --- |
| HHT1ggR | 5’-aaaGGTCTCaATCTcgagggtatcgccaggaaaaac-3’ | Construction of pUC-HHT1-HHF1 |
| HHF1ggR | 5’-aaaGGTCTCaCGACcgatccctattccatgcaagttcg-3’ | Construction of pUC-HHT1-HHF1 |
| HHT2ggR | 5’-aaaGGTCTCaATCTgccggttataaaattacgtatactatctaagc-3’ | Construction of pUC-HHT2-HHF2 |
| HHF2ggR | 5’-aaaGGTCTCaCGACcggcaatgaaatggaggagc-3’ | Construction of pUC-HHT2-HHF2 |
| hht1F | 5’-ctttctagttaataagaaaaacatctaacataaatatataaacgcaaacaATGGCTAGGACCAAACAGACTGC-3’ | PCR primer for generating donors to replace *HHT1* with *synH3* |
| hht1R | 5’-gtgtttttgttcgttttttactaaaactgatgacaatcaacaaaCTAGCTCCTCTCGCCACGCAG-3’ | PCR primer for generating donors to replace *HHT1* with *synH3* |
| hht2F | 5’-cttccaactgttcttccccttttactaaaggatccaagcaaacactccacaATGGCTAGGACCAAACAGACTGC-3’ | PCR primer for generating donors to replace *HHT2* with *synH3* |
| hht2R | 5’-gtccccccagtctaaatgcatagaaaaaaaaaaattcccgctttatatCTAGCTCCTCTCGCCACGCAG-3’ | PCR primer for generating donors to replace *HHT2* with *synH3* |
| hhf1F | 5’-gtactaaagcaacaaacaaaaacaagcaacaaatataatatagtaaaatATGTCTGGAAGGGGAAAGGG-3’ | PCR primer for generating donors to replace *HHF1* with *synH4* |
| hhf1R | 5’-gaatcccaaatatttgcttgttgttaccgttttcttagaattagctaaaTTACCCTCCAAAGCCGTACAATGTAC-3’ | PCR primer for generating donors to replace *HHF1* with *synH4* |
| hhf2F | 5’-tacatcttgttcaaaagagtagcaaaaacaacaatcaatacaataaaataATGTCTGGAAGGGGAAAGGG-3’ | PCR primer for generating donors to replace *HHF2* with *synH4* |
| hhf2R | 5’-gaaaggcatgaaaataatttcaaacaccgattgtttaaccaccgattgtTTACCCTCCAAAGCCGTACAATGTAC-3’ | PCR primer for generating donors to replace *HHF2* with *synH4* |
| hht1k4rR | 5’-ccagtggactttcttgctgtttgTCTtgttctggccattgtttgcgtttatatatttatg-3’ | PCR primer for generating donors to replace *synH3* with *hht1* K4R |
| hht1k4rF | 5’-cataaatatataaacgcaaacaatggccagaacaAGAcaaacagcaagaaagtccactgg-3’ | PCR primer for generating donors to replace *synH3* with *hht1* K4R |
| hht2k4rR | 5’-ctttaccaccagtggattttctagctgtttgTCTagttctggccattgtggagtgtttgc-3’ | PCR primer for generating donors to replace *synH3* with *hht2* K4R |
| hht2k4rF | 5’-gcaaacactccacaatggccagaactAGAcaaacagctagaaaatccactggtggtaaag-3’ | PCR primer for generating donors to replace *synH3* with *hht2* K4R |
| hht12k36rR | 5’-gtacctggcttatatctgtgaggcttTCTaacaccaccggtagatggggcggattttc-3’ | PCR primer for generating donors to replace *synH3* with *hht1/2* K36R |
| hht12k36rF | 5’-gaaaatccgccccatctaccggtggtgttAGAaagcctcacagatataagccaggtac-3’ | PCR primer for generating donors to replace *synH3* with *hht1/2* K36R |
| hht12k79rR | 5’-gcagaagattgaaatctcaagtcggtTCTgaaatcttgagcgatttctctgaccaatc-3’ | PCR primer for generating donors to replace *synH3* with *hht1/2* K79R |
| hht12k79rF | 5’-gattggtcagagaaatcgctcaagatttcAGAaccgacttgagatttcaatcttctgc-3’ | PCR primer for generating donors to replace *synH3* with *hht1/2* K79R |
| HHT1HRF | 5’-ctggccgtaatatctctccttgac-3’ | PCR primer for generating donors to replace *synH3* with *hht1* |
| HHT1HRR | 5’-cgagggtatcgccaggaaaaac-3’ | PCR primer for generating donors to replace *synH3* with *hht1* |
| HHT2HRF | 5’-cgcccaatgtgacgcacaatc-3’ | PCR primer for generating donors to replace *synH3* with *hht2* |
| HHT2HRR | 5’-gccggttataaaattacgtatactatctaagc-3’ | PCR primer for generating donors to replace *synH3* with *hht2* |
| HHF1HRF | 5’-cgcgaatacggtggtaaattgc-3’ | PCR primer for generating donors to replace *synH4* with *hhf1* |
| HHF1HRR | 5’-cgatccctattccatgcaagttcg-3’ | PCR primer for generating donors to replace *synH4* with *hhf1* |
| HHF2HRF | 5’-cactcgcgcctgggctgttg-3’ | PCR primer for generating donors to replace *synH4* with *hhf2* |
| HHF2HRR | 5’-cggcaatgaaatggaggagc-3’ | PCR primer for generating donors to replace *synH4* with *hhf2* |

**Supplementary table S2. Primers for Construction of pCas Vectors**

| **Primer** | **Sequence** | **Function** |
| --- | --- | --- |
| C9h3.1F | 5’-AAAGGTCTCAGATCagaaaatccgccccatctacGTTTTAGAGCTAGAAATAGCAAGTTA-3’ | gRNA1 Targeting *HHT1/2* |
| C9h3.1R | 5’-AAAGGTCTCTAAACaaaatctactgaactgttgaTGCGCAAGCCCGGAATCGAACCGGG-3’ | gRNA2 Targeting *HHT1/2* |
| C9h4.1F | 5’-AAAGGTCTCAGATCggtggtgtcaagcgtatttcGTTTTAGAGCTAGAAATAGCAAGTTA-3’ | gRNA1 Targeting *HHF1/2* |
| C9h4.1R | 5’-AAAGGTCTCTAAACagcgtcacagaaagattctaTGCGCAAGCCCGGAATCGAACCGGG-3’ | gRNA2 Targeting *HHF1/2* |
| C9m3.1F | 5’-AAAGGTCTCAGATCCAGGAAACAGTTGGCCTCCAGTTTTAGAGCTAGAAATAGCAAGTTA-3’ | gRNA1 Targeting *synH3* |
| C9m3.1R | 5’-AAAGGTCTCTAAACCCGAGCTACTAATAAGGAAATGCGCAAGCCCGGAATCGAACCGGG-3’ | gRNA2 Targeting *synH3* |
| C9m4.1F | 5’-AAAGGTCTCAGATCATATTGAGGGACAATATTCAGTTTTAGAGCTAGAAATAGCAAGTTA-3’ | gRNA1 Targeting *synH4* |
| C9m4.1R | 5’-AAAGGTCTCTAAACCCCCTACGTGCTAGACGACGTGCGCAAGCCCGGAATCGAACCGGG-3’ | gRNA2 Targeting *synH4* |
| C9negF | 5’-AAAGGTCTCAGATCGTTTTAGAGCTAGAAATAGCAAGTTA-3’ | negative control plasmid (no gRNA) |
| C9negR | 5’-AAAGGTCTCTAAACTGCGCAAGCCCGGAATCGAACCGGG-3’ | negative control plasmid (no gRNA) |
| H1m1F | 5’-AAAGGTCTCAGATCCAGGAAACAGTTGGCCTCCAGTTTTAGAGCTAGAAATAGCAAGTTA-3’ | gRNA1 Targeting *synH3* (PCR from psgtRNA) |
| H1m1R | 5’-AAAGGTCTCAGTCCCTCAATATTGCGCAAGCCCGGAATCGAACCGGG-3’ | gRNA1 Targeting *synH4* (PCR from psgtRNA) |
| H1m2F | 5’-AAAGGTCTCAGGACAATATTCAGTTTTAGAGCTAGAAATAGCAAGTTAA-3’ | gRNA1 Targeting *synH4* (PCR from pscURA3) |
| H1m2R | 5’-AAAGGTCTCATGCTAGACGACGGATCATTTATCTTTCACTGCGG-3’ | gRNA2 Targeting *synH4* (PCR from pscURA3) |
| H1m3F | 5’-AAAGGTCTCAAGCACGTAGGGGGTTTTAGAGCTAGAAATAGCAAGTTAA-3’ | gRNA2 Targeting *synH4* (PCR from psgtRNA) |
| H1m3R | 5’-AAAGGTCTCTAAACCCGAGCTACTAATAAGGAAATGCGCAAGCCCGGAATCGAACCGGG-3’ | gRNA2 Targeting *synH3* (PCR from psgtRNA) |

**Supplementary table S3. Primers for Sequencing**

| **Primer** | **SeQUENCE** | | **Function** |
| --- | --- | --- | --- |
| HHF2SeqF | | 5’-ccagaacaaaacaactgacaagaagg-3’ | sequencing primer |
| HHF2SeqR | | 5’-ctgcttaaccaacagagggg-3’ | sequencing primer |
| HHT2SeqF | | 5’-gcgtgaaactattggcacgcc-3’ | sequencing primer |
| HHT2SeqR | | 5’-cctttacgtaactagtctcttcgaaaatg-3’ | sequencing primer |
| HHF1SeqF | | 5’-cgagcgcttctccccataatgg-3’ | sequencing primer |
| HHF1SeqR | | 5’-cgaggcgcgtgtaagttacagac-3’ | sequencing primer |
| HHT1SeqF | | 5’-gtcccatacattatcgttctcacaatttc-3’ | sequencing primer |
| HHT1SeqR | | 5’-ccacatggaaagccataaatcttgc-3’ | sequencing primer |

**Supplementary Note:**

In order to eliminate the possibility that the donor DNA fragments would be integrated into other chromosomal positions with random insertion, the whole genome sequencing of *synH3H4* strain was performed by Annoroad Gene Technology (Beijing) Co., Ltd.. The sequencing was analyzed through method described in Schneeberger K, et al *PNAS*. 2011 Jun 21;108(25):10249-54. The whole genome sequencing results were assembled into 451 scaffolds. We searched whole scaffolds for synH3 5’ 20bp partial sequence “ATGGCTAGGACCAAACAGAC”, synH3 3’ sequence “TGCGTGGCGAGAGGAGCTAG”, synH4 5’ sequence “ATGTCTGGAAGGGGAAAGGG”, and synH4 3’ sequence “TGTACGGCTTTGGAGGGTAA”, and all results came only 2 hits: scaffold 70 and 199. After analyzing these two scaffolds, we identified they are corresponding to *HHT* and *HHF* genes on Chr. II and Chr. XIV. However, we did not find these synH3 or synH4 partial sequences aligned to any other scaffolds which demonstrated that the donor fragment could not integrated into other chromosomal positions with random insertion.

Sequence alignment on scaffold 70 (Chr. II)

1^st^ alignment sequence is the whole genome sequencing results of *SynH3H4* strain; 2^nd^ alignment sequences are *SynH3* or *SynH4*, showing the whole genome sequencing results are identical to the synthetic sequence. 3^rd^ alignment sequence is wild-type references genome sequence from SGD website (<https://www.yeastgenome.org/>). The sequences were aligned using Snapgene software.


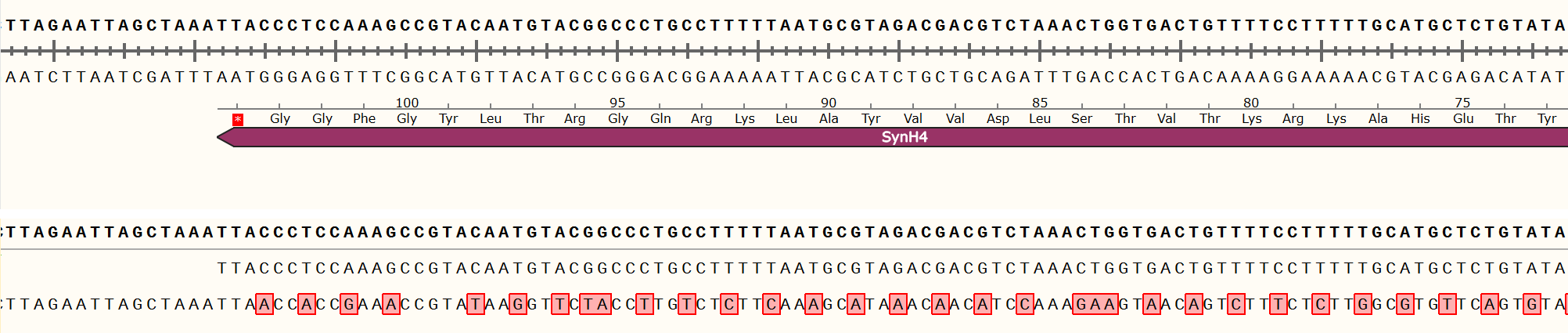


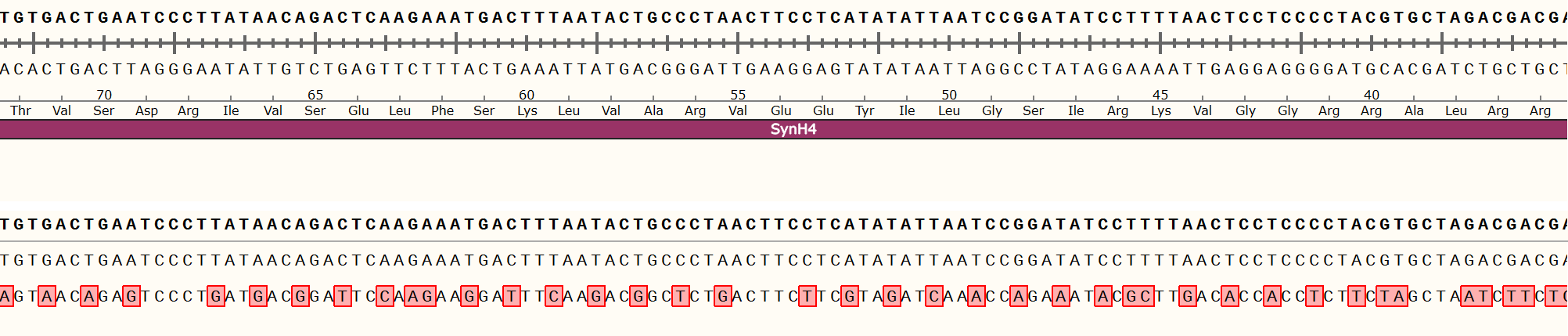


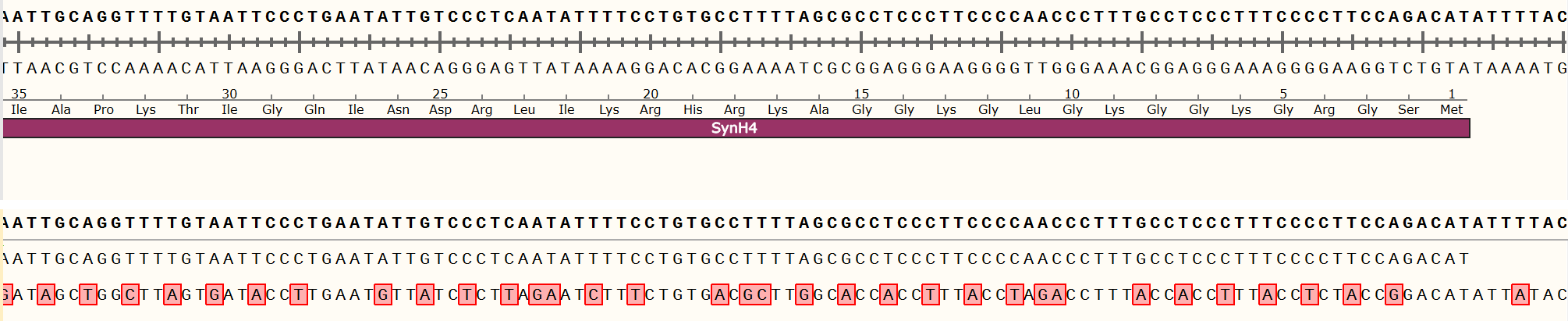


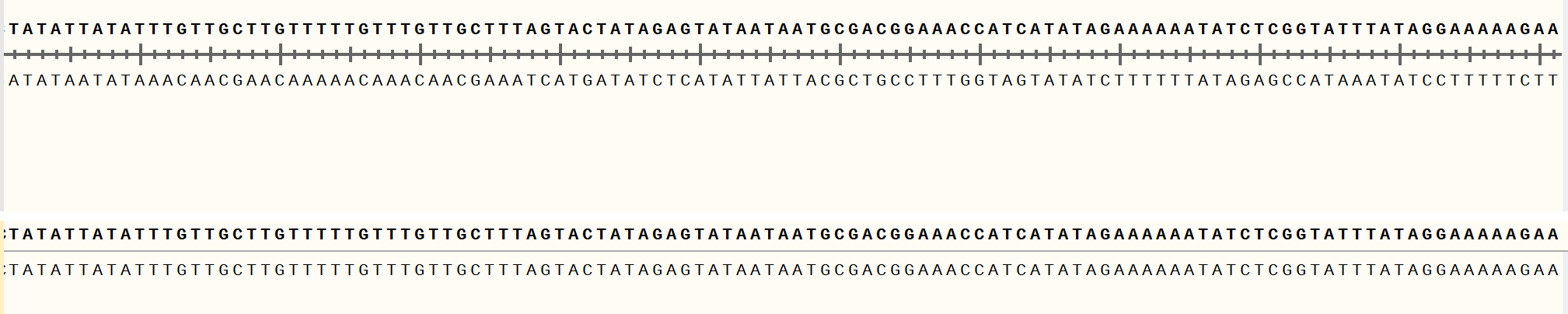


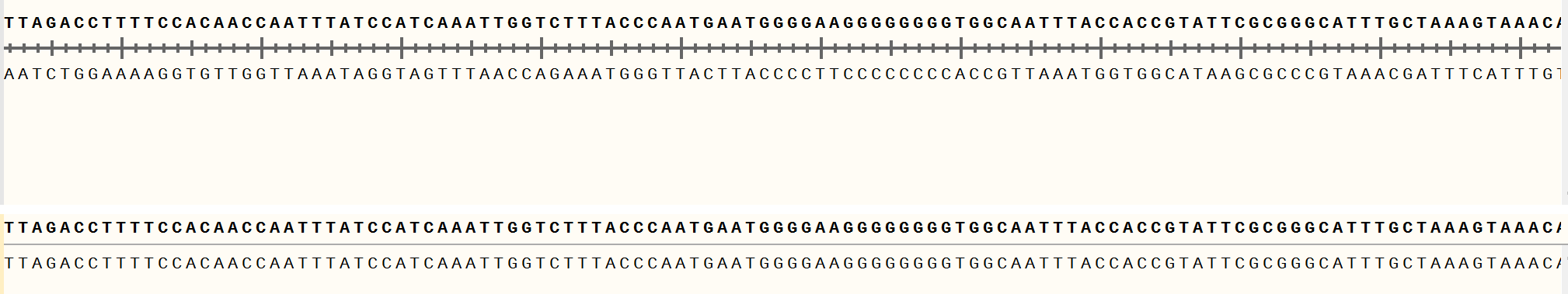


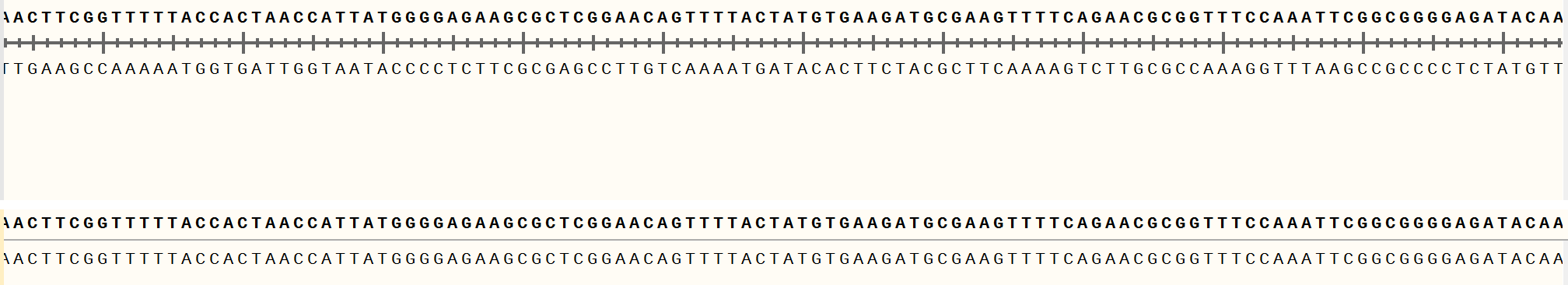


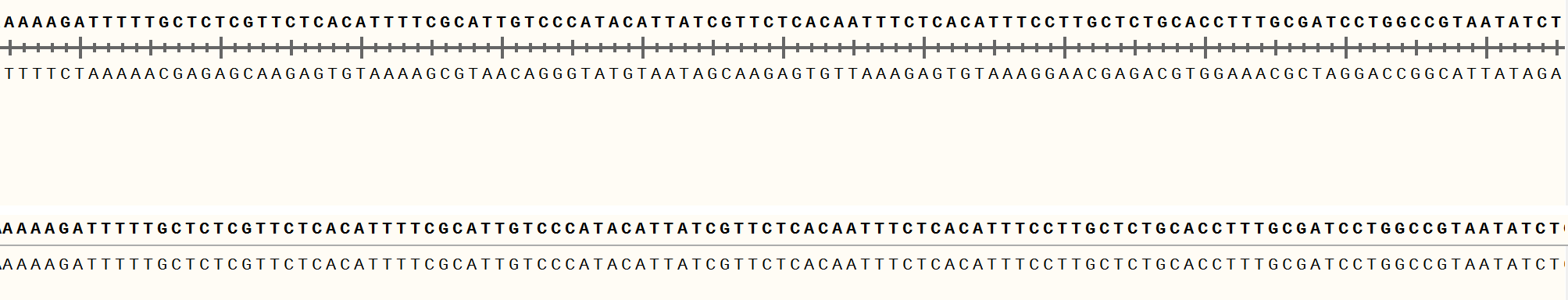


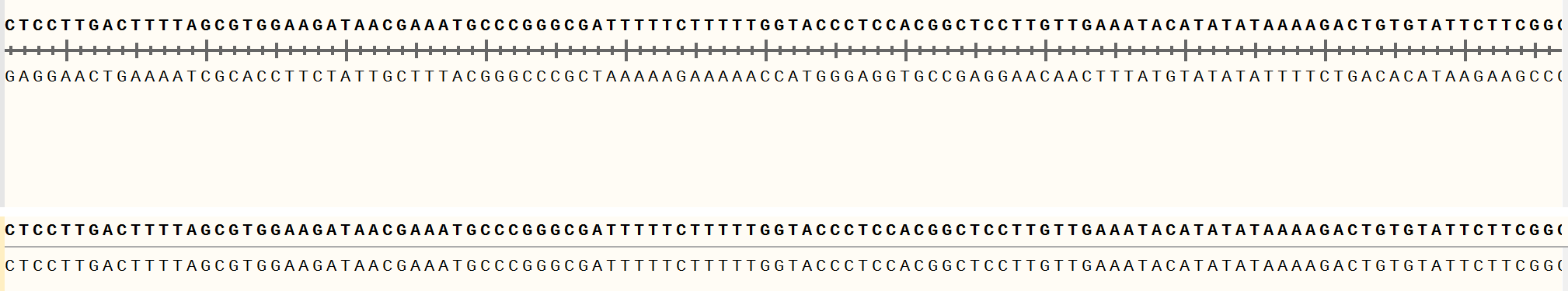


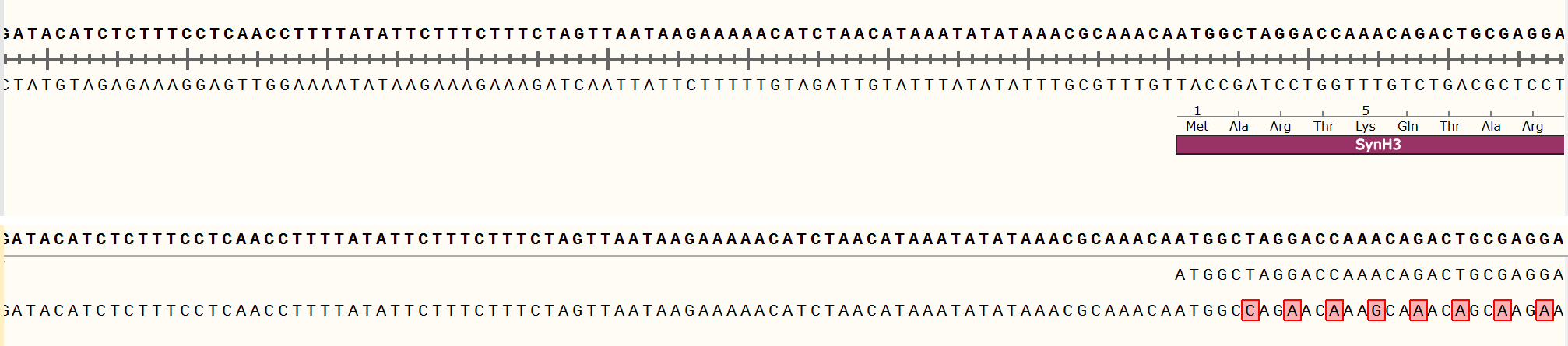


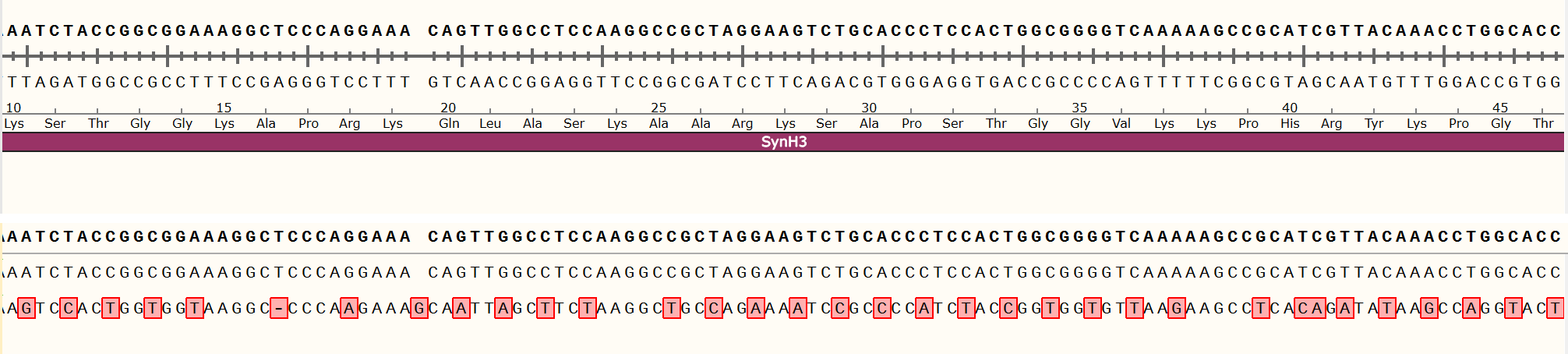


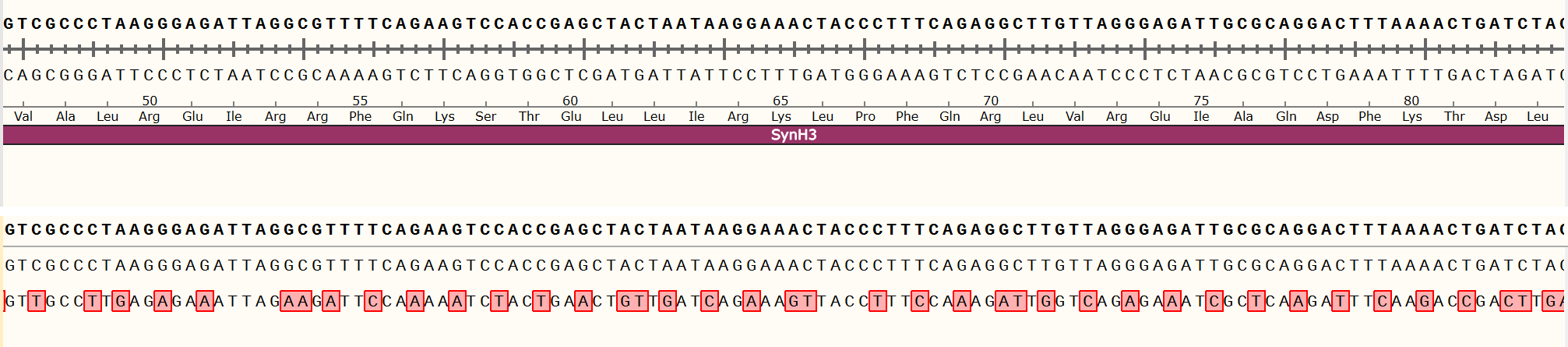


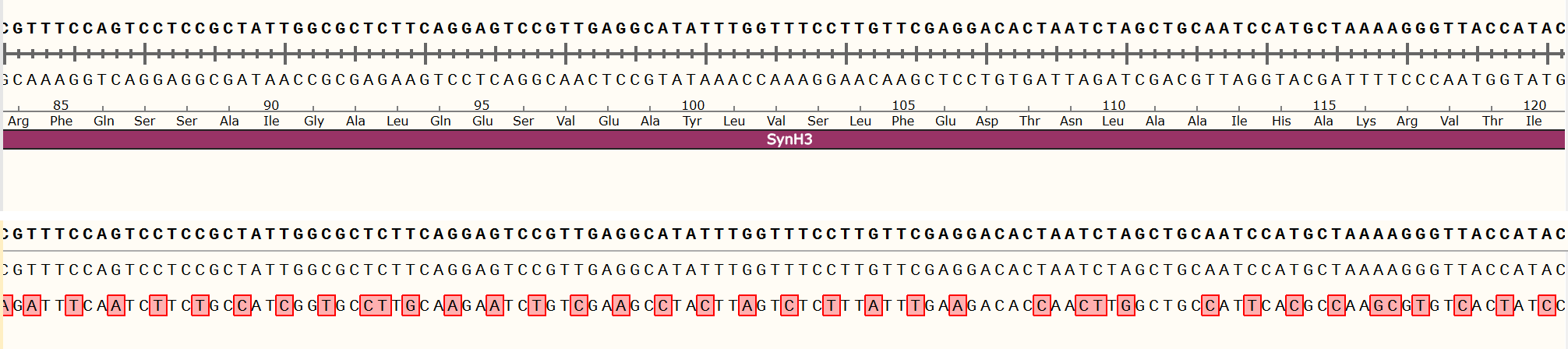


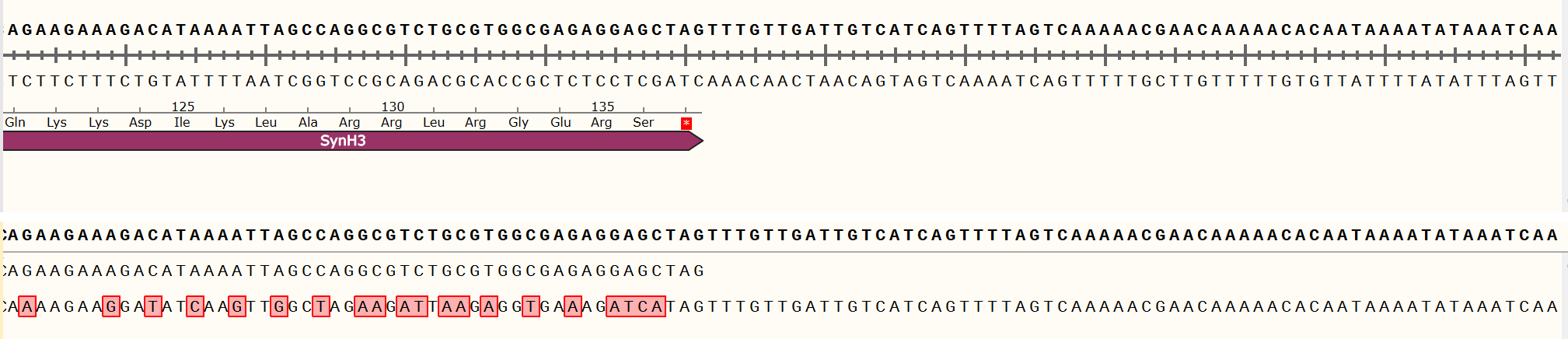


**Sequence alignment on scaffold 199 (Chr. XIV)**


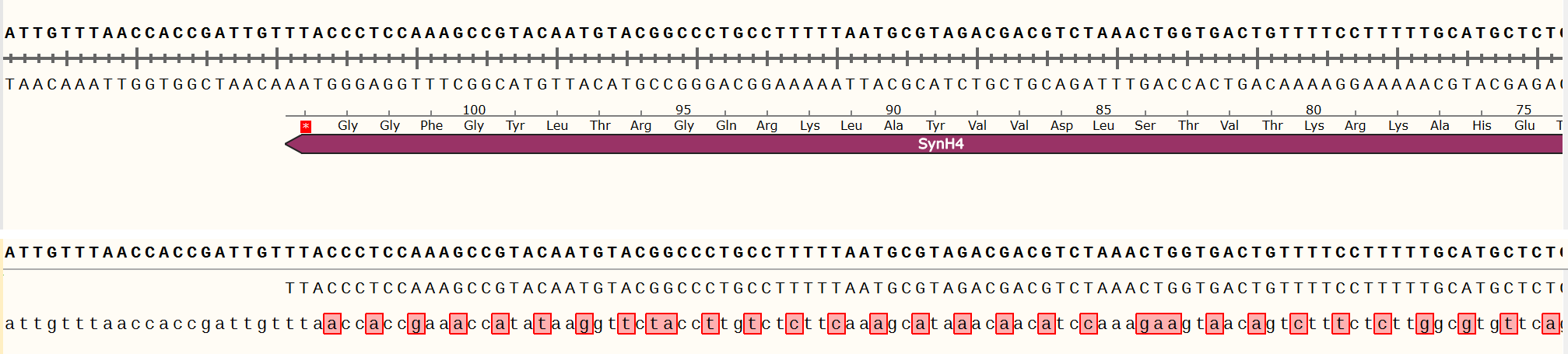

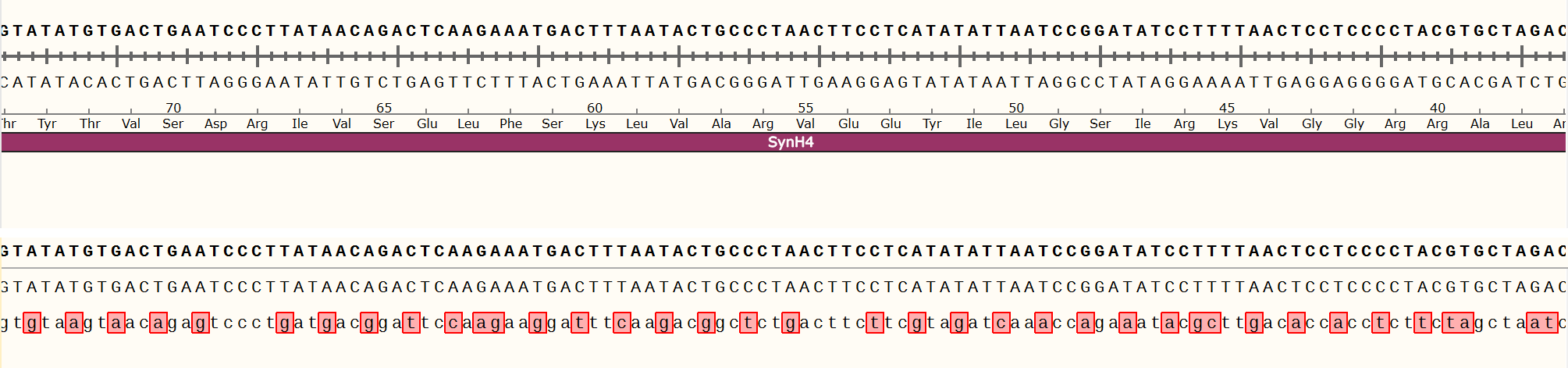

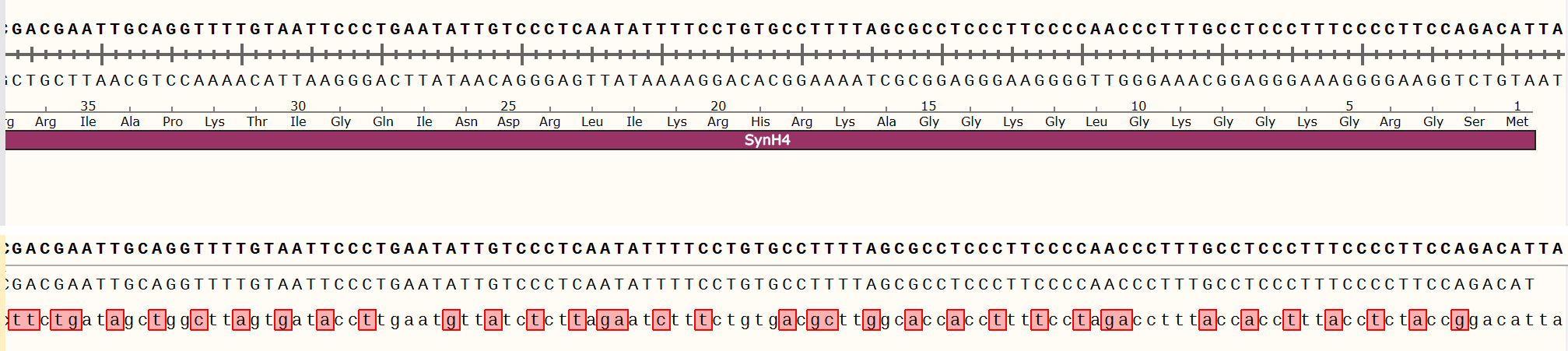

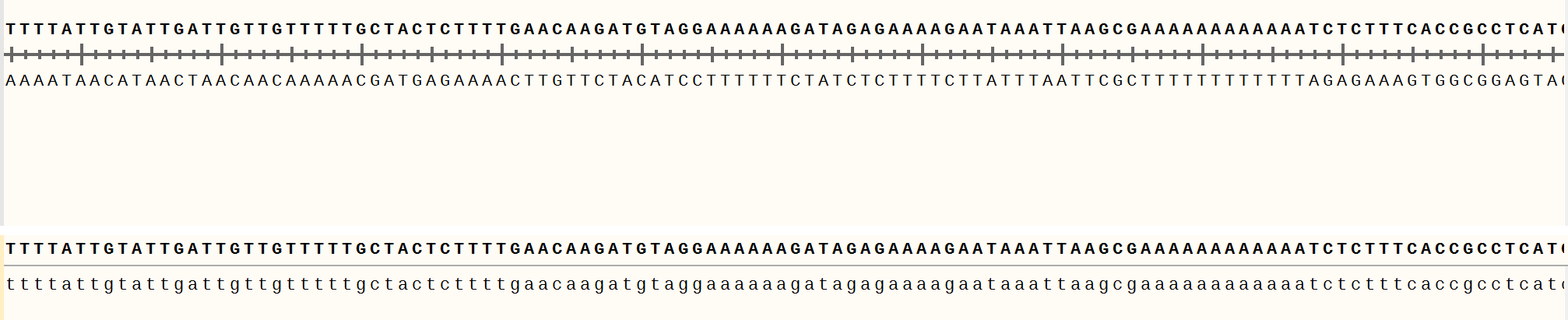

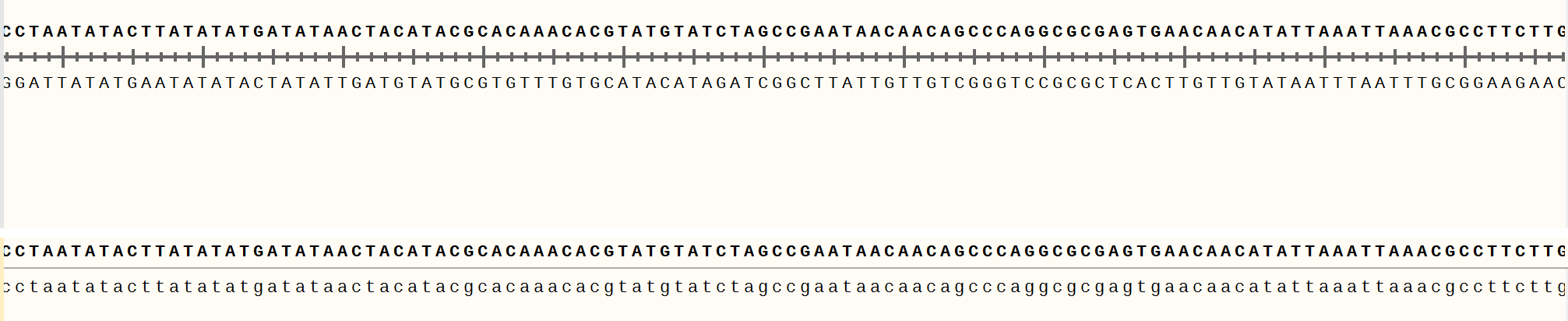


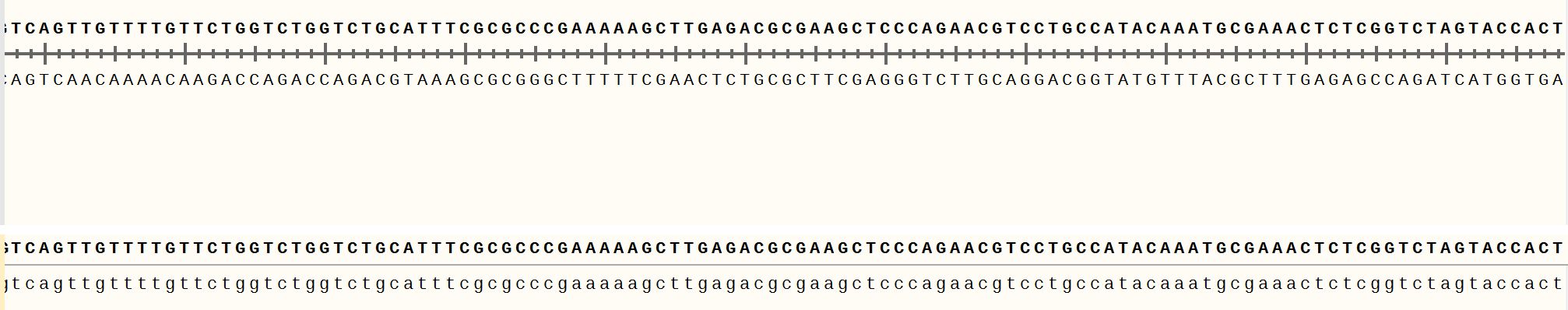

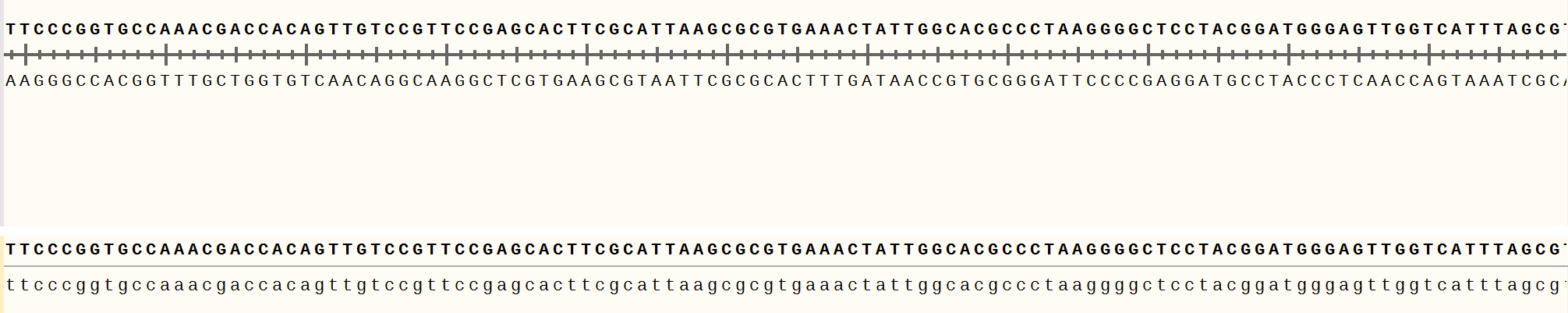


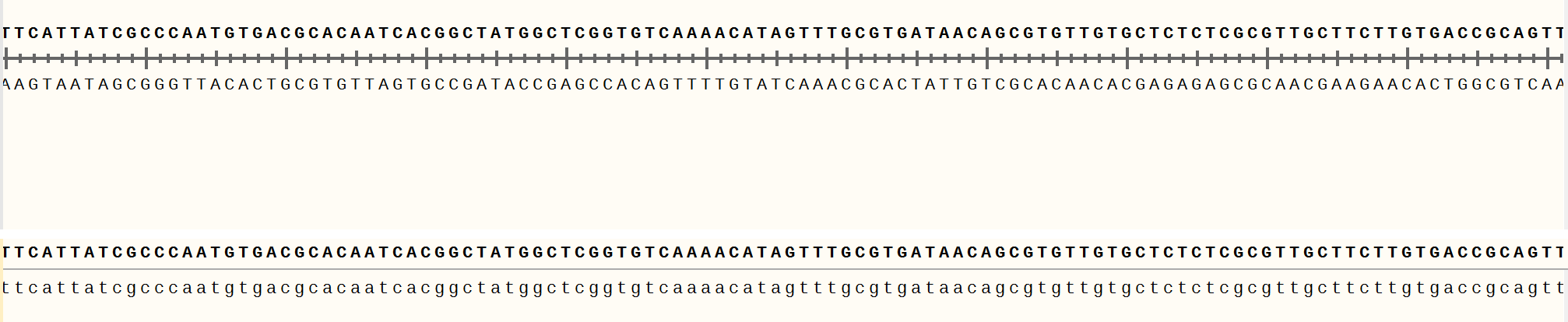

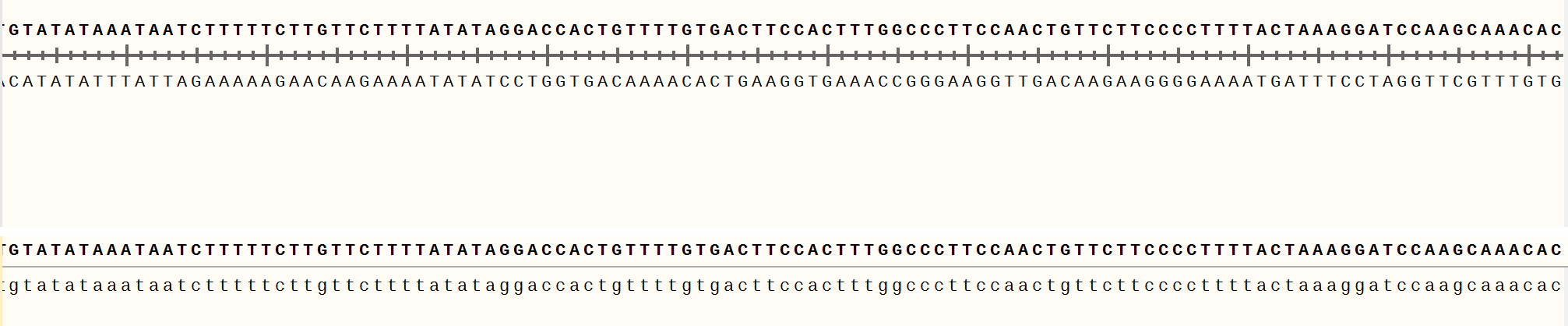

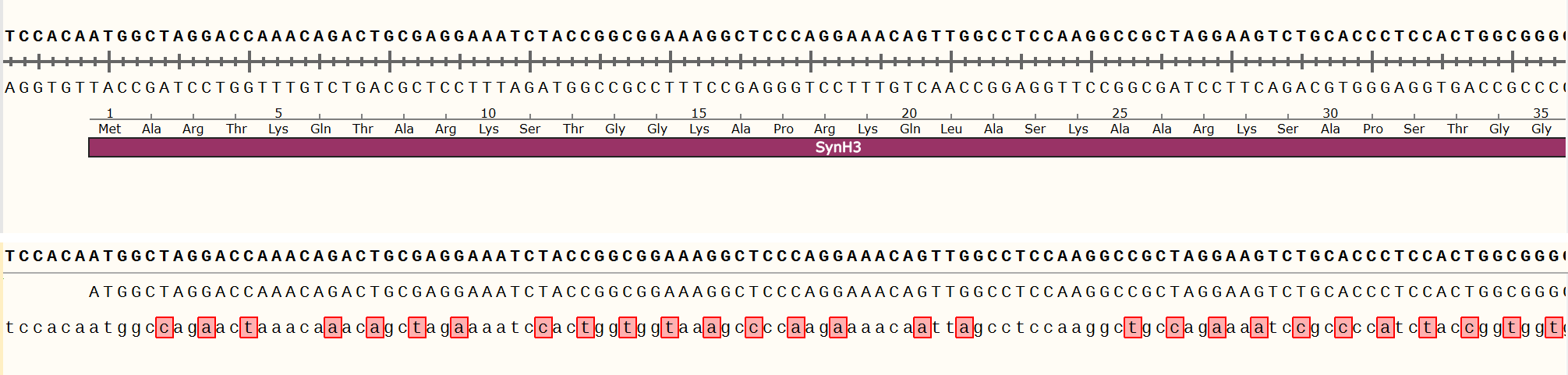

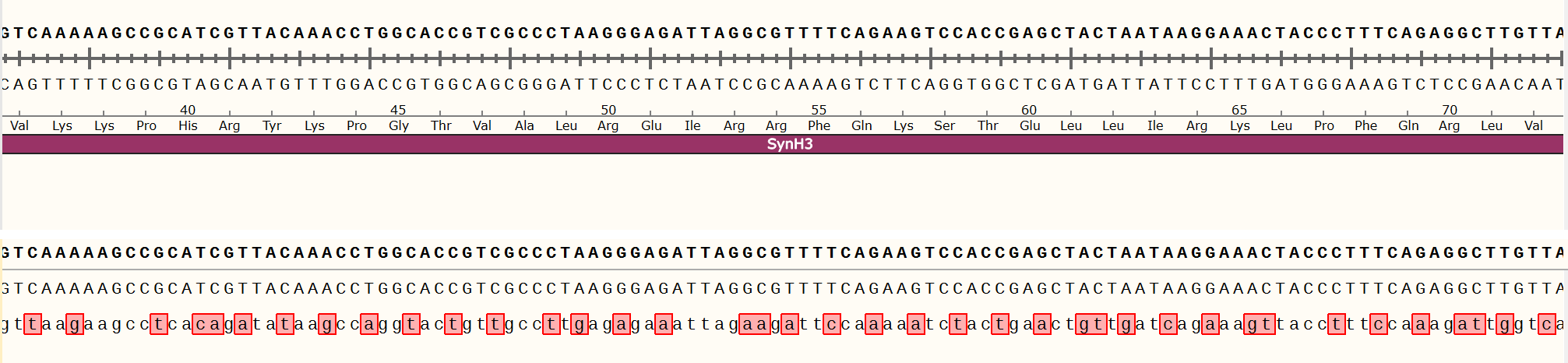

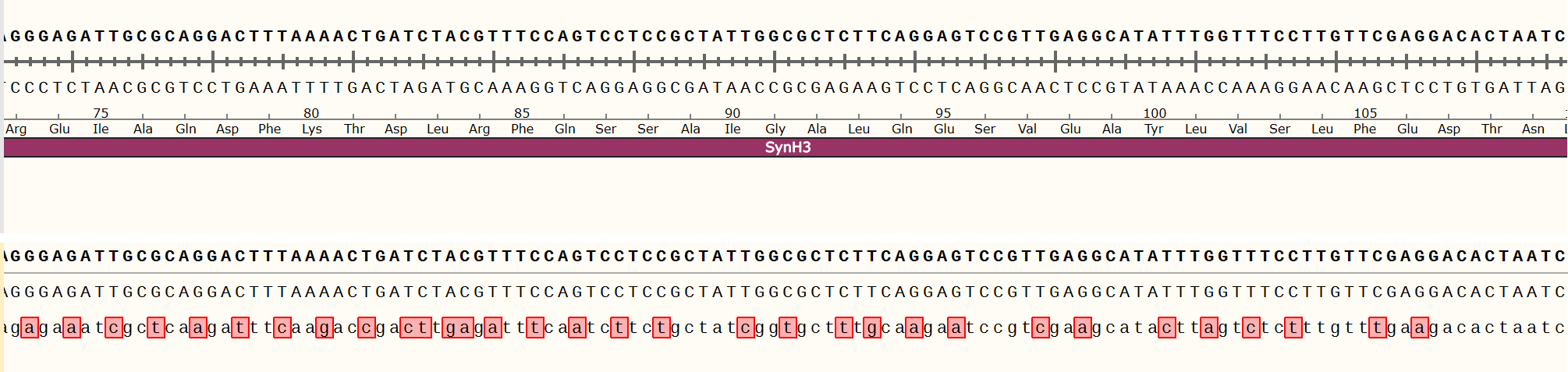

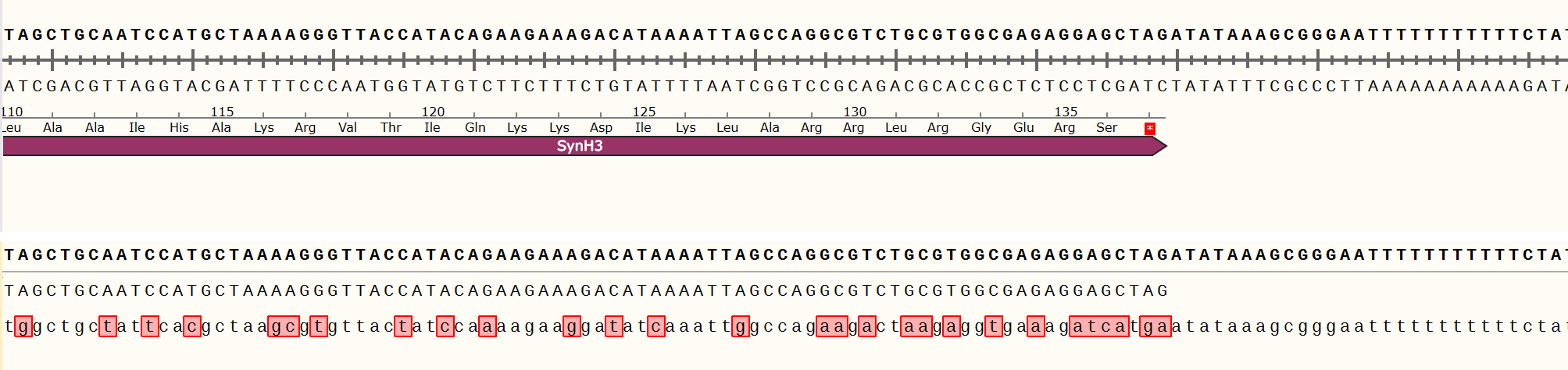

Supplement: Supplementary file 1 — Supplementary Information [file 41598_2021_82774_MOESM1_ESM.docx]
